# Regulation of Redox Profile and Genomic Instability by Physical Exercise Contributes to Neuroprotection in Mice with Experimental Glioblastoma

**DOI:** 10.3390/antiox12071343

**Published:** 2023-06-26

**Authors:** Luis F. B. Marqueze, Amanda K. Costa, Giulia S. Pedroso, Franciane F. Vasconcellos, Bruna I. Pilger, Schellen Kindermann, Vanessa M. Andrade, Ana C. B. Alves, Tatyana Nery, Aderbal A. Silva, Stephanie R. S. Carvalhal, Matheus F. Zazula, Katya Naliwaiko, Luiz C. Fernandes, Zsolt Radak, Ricardo A. Pinho

**Affiliations:** 1 Graduate Program in Health Sciences, School of Life Sciences and Medicine, Pontifical Catholic University of Paraná, Curitiba 80215-200, Brazil; amanda.kruger@pucpr.edu.br (A.K.C.); giulia.fidelis@pucpr.br (G.S.P.); franciane.falcao@pucpr.edu.br (F.F.V.); bruna.pilger@pucpr.edu.br (B.I.P.); 2 Graduate Program in Health Sciences, University of Southern Santa Catarina, Criciúma 88806-000, Brazil; schekindermann@hotmail.com (S.K.); vma@unesc.net (V.M.A.); 3 Department of Physical Therapy, Federal University of Santa Catarina, Araranguá 88905-120, Brazil; ana.cristina.alves@posgrad.ufsc.br (A.C.B.A.); tatyana.nery@posgrad.ufsc.br (T.N.);; 4 Department of Physiology, Federal University of Parana, Curitiba 81531-970, Brazil; stephanie.carvalhal@ufpr.br (S.R.S.C.); matheus.zazula@ufpr.br (M.F.Z.); katya@ufpr.br (K.N.); lcfer@ufpr.br (L.C.F.); 5 Research Institute of Sport Science, University of Physical Education, Alkotas u. 44, H-1123 Budapest, Hungary; radak.zsolt@tf.hu

**Keywords:** physical exercise, glioblastoma, oxidative stress, genomic stability, brain tumor

## Abstract

Glioblastoma (GBM) is an aggressive, common brain cancer known to disrupt redox biology, affecting behavior and DNA integrity. Past research remains inconclusive. To further understand this, an investigation was conducted on physical training’s effects on behavior, redox balance, and genomic stability in GBMA models. Forty-seven male C57BL/6J mice, 60 days old, were divided into GBM and sham groups (*n* = 15, *n* = 10, respectively), which were further subdivided into trained (Str, Gtr; *n* = 10, *n* = 12) and untrained (Sut, Gut; *n* = 10, *n* = 15) subsets. The trained mice performed moderate aerobic exercises on a treadmill five to six times a week for a month while untrained mice remained in their enclosures. Behavior was evaluated using open-field and rotarod tests. Post training, the mice were euthanized and brain, liver, bone marrow, and blood samples were analyzed for redox and genomic instability markers. The results indicated increased latency values in the trained GBM (Gtr) group, suggesting a beneficial impact of exercise. Elevated reactive oxygen species in the parietal tissue of untrained GBM mice (Gut) were reduced post training. Moreover, Gtr mice exhibited lower tail intensity, indicating less genomic instability. Thus, exercise could serve as a promising supplemental GBM treatment, modulating redox parameters and reducing genomic instability.

## 1. Introduction

Cancers of the central nervous system (CNS) are ranked 14th among men (estimated risk, 3.9/100,000) and 15th among women (estimated risk, 3.0/100,000) among all cancer cases. Although CNS cancers are relatively rare, they significantly contribute to mortality worldwide [1]. Among the malignant neoplasms that reach the CNS, those that affect glial cells (gliomas) are prominent. These gliomas account for 81% of all primary CNS tumors in adults in the United States [2]. Gliomas are tumors of the cerebral parenchyma that are histologically classified based on their similarity to different types of glial cells. Their degree of aggressiveness varies with the growth and invasion of adjacent tissues and is classified as I, with lower intensity, and IV, with higher intensity.

Glioblastoma (GBM) is the most recurrent astrocytoma in adults, with high aggressiveness and invasive behavior. GBM has a clinical course demarcated by palliative protocols and treatments, with a life expectancy of approximately 10–15 months after diagnosis. The clinical course of GBM is influenced by its location and infiltration rate, which are characterized by intense deleterious processes involving tissue destruction, edema, and epilepsy. Individuals with GBM often have severe mood swings, which are commonly confused with psychotic episodes, in addition to motor sensory impairment [3].

Nonpharmacological therapies are complementary options that can contribute to GBM treatment, because their benefits have already been proven in different neuropathologies. Among nonpharmacological therapies, physical exercise has received greater prominence for its proven efficiency in different neoplasms but lacks a description of its effects in the context of patients with GBM [4,5,6,7]. Lemke et al. [8] reported that combining temozolomide and exercise reduced intracellular levels of reactive oxygen species (ROS) in glioma cells, because they improved intratumoral vascularization and consequently increased the efficiency of medications. Therefore, the biochemical and molecular bases of diseases associated with new therapeutic approaches should be investigated.

Regular physical exercise is an important nonpharmacological intervention, because it has already demonstrated significant changes in the pathophysiology of several other types of cancer. Therefore, physical exercise has been proposed as a nonpharmacological resource with physiological implications for neuroprotection and attenuation of tumor progression. This study aimed to determine the role of physical exercise in GBM development.

## 2. Material and Methods

### 2.1. Animals and Bioethical Procedures

Forty-seven male C57BL/6J mice aged sixty days were randomly selected and divided into two groups (GBM and sham/placebo surgery), which were subsequently divided into four groups: untrained sham (Sut, *n* = 10), untrained GBM (Gut, *n* = 15), trained sham (Str, *n* = 10), and trained GBM (Gtr, *n* = 12). The mice were purchased from Carlos Chagas Institute, Paraná, Brazil, and maintained with a 12–12 h light–dark cycle at a temperature of 22 ± 1 °C and with free access to water and food. All experimental procedures were performed according to the Animal Research: Reporting of In Vivo Experiments 2.0 guidelines [9] and Brazilian Guidelines for the Use of Animals and were approved by the Ethics Committee for the Use of Animals of the Federal University of Paraná (protocol number: 23075.041588/2019-00).

### 2.2. Training Protocol

One week after GBM induction, the animals were placed on a motorized rat treadmill (Scienlabor 6 SLP 033 Bay) for three 10 min sessions per day at a speed of 6 m/min. Four animals from each group underwent a progressive exercise test to determine their maximum speed, starting at 10 m/min and increasing at a rate of 3 m/min every 2 min. The test was terminated when the animal was exhausted or when it reached a speed of 30 m/min. A training program, adapted from Souza et al. [10], was implemented for 4 weeks, with five to six sessions per week and a rest day every three sessions. The treadmill speed progressed from 50 to 60% of the predetermined maximal speed, and each session lasted for up to 50 min without inclination. Noxious stimuli were not used to encourage running. The untrained groups were kept in their cages throughout the experimental period.

### 2.3. Experimental Glioblastoma Protocol

GL261 cells were obtained from the Rio de Janeiro Cell Bank, thawed, and cultured for 1 week in high-glucose Dulbecco’s Modified Eagle Medium containing 10% fetal bovine serum (Thermo Fisher Scientific, Waltham, MA, USA) to achieve 80–90% confluency. Intracerebroventricular administration of 3 μL (2 × 10^5^ cells/mL) of GL261 cells was performed under anesthesia (110 mg/kg ketamine and 10 mg/kg xylazine) using a stereotactic procedure at the following coordinates: 2 mm to the right of the midline and 1 mm to the front of the bregma to a depth of 2.5 mm. Animals in the sham group received 3 μL of phosphate-buffered saline at the same coordinates [11]. Postoperatively, the animals received tramadol 5 mg/kg 1 h after awakening and every 8 h for 2 consecutive days to manage pain.

### 2.4. Body Mass, Water, and Food Control

The average body weight of the animals was determined weekly using a 0.001 g Filizola precision scale. Feed intake was controlled by measuring the average weight of feed deposited in the boxes. Net intake was determined by calculating the average amount of feed ingested daily by the animals.

### 2.5. Rotarod Test

The motor coordination of the animals was assessed by the latency to fall in the rotarod tests [12]. The apparatus consists of a rotating rod (3.7 cm in diameter) divided into four separate compartments, placed at a height of 25 cm, and rotated at a fixed speed of 8 rpm. The latency to fall was also quantified. The animals were tested before induction surgery and every 7 days until day 28 in the groups without training and only before and on the seventh day after surgery and before euthanasia.

### 2.6. Open-Field Test

This test assesses the general level of locomotor activity, exploration, and anxiety. The animal was placed in a square box (42 × 42 × 42 cm^3^), in the center of which a smaller square was drawn for a period of 5 min. A camera was used to monitor movement in and around the central and peripheral areas of the box. During this period, the number of times each mouse relied only on its hind legs (rearing), number of times it crossed the square drawn in the center of the box used (crossing), length of time the animal remained in the center of the quadrant (duration in the quadrant), and number of times it returned to the quadrant (number of entries into the quadrant) were counted [13,14].

### 2.7. Euthanasia and Sample Preparation

The animals were euthanized by cervical displacement for 48 days (72 h after the last training session). Samples from the prefrontal cortex, parietal lobe (D/E), and right quadriceps were collected and immediately aliquoted into tubes without additives and stored in liquid nitrogen (down to −140 °C) and then stored in a freezer (−80 °C) until biochemical assays were completed. In the bone marrow of the right thigh, 1 mL of blood (obtained by bleeding the neck) and a piece of the liver were stored in heparinized microtubes and kept refrigerated for further DNA damage analysis. The remaining animal tissues and carcasses were placed in a milky white bag and stored in a freezer until they were collected and transported for final disposal in an appropriate landfill. All procedures were performed in accordance with the Guideline for the Practice of Euthanasia of the National Council for Animal Control and Experimentation, Brazil

### 2.8. Redox Parameter Assays

A spectrophotometer (VersaMax ELISA) was used to evaluate the redox parameters. The Amplex^®^ Red A22188 kit (Invitrogen, Paisley, UK) was used to measure hydrogen peroxide (H_2_O_2_) production, with the reagent 10-acetyl-3,7-dihydroxyphenoxazine (Amplex^®^ red) reacting in a 1:1 stoichiometry with H_2_O_2_ BTO producing highly fluorescent analyte resorufin. Samples were homogenized in a special buffer and incubated with the reagent for 30 min. After incubation, absorbance was measured at 560 nm. To determine the carbonylated amino acid residues, the samples were first homogenized in a special buffer at room temperature and then washed successively with 20% trichloroacetic acid, 70% ethanol, and ethyl acetate (1:1) to add urea (8 M). The reaction of the carbonyl groups in the sample with 2,4-dinitrophenylhydrazine was measured at 366 nm. Carbonyl content was determined using a molar extinction coefficient of 22,000 M [15]. The malondialdehyde (MDA) content was determined using the MAK085 kit (Sigma Aldrich, St. Louis, MO, USA) by reacting MDA with thiobarbituric acid to obtain a colorimetric reaction reading at 532 nm. The total protein content was measured in all samples to normalize the results obtained in each assay using an optimized Bradford method [16]. Albumin was used as the standard protein, and linear regression results were used to determine the total protein concentration (mg).

### 2.9. Comet Test

The comet test was conducted under alkaline conditions [17]. The tissues were collected in heparinized and refrigerated microtubes. Cells (5 μL aliquots) were placed in low-melting-point agarose (0.75%, *w*/*v*, 95 μL and 75 μL, respectively). The mixture was placed on a slide previously covered with normal-melting-point agarose (1.5%), then covered with a lamin, and placed in the refrigerator for approximately 5 min at 4 °C to solidify. Briefly, the lamins were carefully removed, and the slides were placed in lysis buffer (2.5 M of sodium chloride, 100 M of ethylenediamine tetraacetic acid (EDTA), and 10 M of Tris, pH 10.0–10.5, with the addition of 1% Triton X-100 and 10% dimethyl sulfoxide) at 4 °C for a minimum of 1 h and a maximum of 1 week. The slides were incubated in alkaline buffer (300 mM of sodium hydroxide and 1 mM of EDTA, pH > 13) for 20 min for DNA unfolding. The electrophoretic run was performed in the same buffer under the following conditions: at 25 V and 300 mA for 15 min. All steps were performed under dim yellow indirect light. Subsequently, the slides were neutralized with 0.4 M of Tris (pH 7.5), and the DNA was corrected with SYBR Gold (Invitrogen, Waltham, MA, USA) for further analysis. To assess the damage, the slides were visualized under a fluorescence microscope at a magnification of 200× using the Comet Assay IV program, and 500 cells/animal were evaluated. Cells were automatically classified according to the ratio of tail length (distance from the center of the nucleus to the end of the tail in μm) and tail moment (tail length × tail fluorescence intensity) by tail intensity (%). Negative and positive controls were used for each electrophoresis assay to ensure reliability of the procedure. All the slides were coded for blank analysis.

### 2.10. Micronucleus Test

The micronucleus test was performed in accordance with the U.S. Environmental Protection Agency’s Gene Tox Program [18]. After bone marrow collection, a swab was prepared directly on the blade with a drop of fetal bovine serum. The slides were stained with Giemsa 5% and dried for blank analysis. As a measure of bone marrow toxicity, the ratio of polychromatic erythrocytes to normochromatic erythrocytes (PCE/NCE) was analyzed in 500 erythrocytes/animal. The frequency of micronuclei (MN) was observed in 2000 PCEs and NCEs for each animal (1000 per slide, in duplicate) using a white-light optical microscope at 1000× magnification. The arithmetic means of micronucleated polychromatic erythrocytes (MN-PCE) and micronucleated normochromic erythrocytes (MN-NCE) was used as the experimental unit.

### 2.11. Data Analysis

The normal distribution of all analyzed parameters was confirmed using the Shapiro–Wilk test. The data were presented as mean and standard deviation and statistically analyzed using a two-way analysis of variance. For most parameters, the Bonferroni post hoc test was applied, while for the open field (center of square) and rotarod at 21 days, the Kruskal–Wallis test was used, followed by the Dunn post hoc test. The Student *t* test was used to compare sham and GBM groups in the body mass data and behavioral tests. A significance level of *p* < 0.05 was established in the statistical tests to determine the minimum intra- and inter-group differences. The statistical package GraphPad Prism version 8.0 was employed for conducting the analyses.

## 3. Results

### 3.1. Body Mass Control

**Figure 1:**
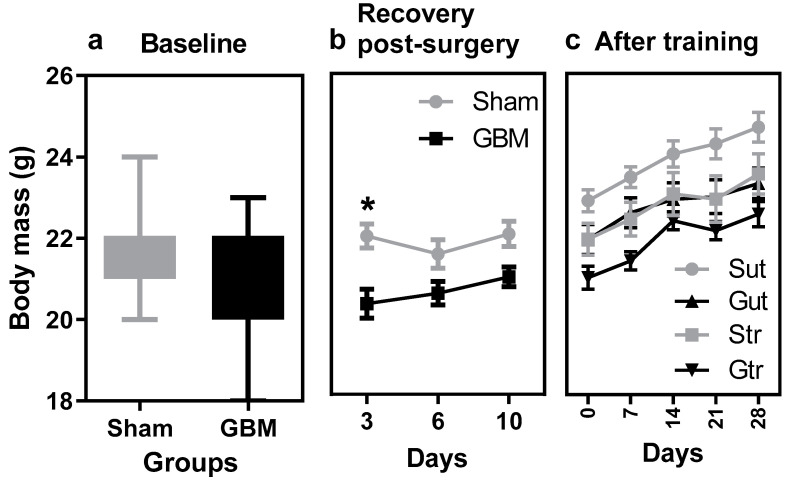
Body mass data from animals subjected to an experimental model of glioblastoma and physical training. (**a**) Mean body mass before surgery, (**b**) mean body mass after surgery, (**c**) mean body mass during and after the training phase. Gut, untrained glioblastoma (*n* = 15); Gtr, trained glioblastoma (*n* = 12); Sut, untrained sham (*n* = 10); Str, trained sham (*n* = 10). * difference between sham versus GBM group.

The findings presented in Figure 1 revealed a distinct pattern of weight changes in response to surgery and training in the two groups under study. Before surgery, no marked differences were observed between the groups (Figure 1a). Subsequently, a lower rate of weight gain was observed in the GBM group compared with the sham group (*p* = 0.0047, 95% CI = 7.615 to 57.30; SE = 8.982), which showed signs of recovery by day 10 (Figure 1b). During the training phase, all groups exhibited changes in body mass; however, no statistically significant differences were observed either within or between the groups (Figure 1c).

### 3.2. Control of Water and Food Consumption

Table 1 shows no significant difference in water and food intake among groups at the start of the study; however, by the end of this study, all groups, except the untrained GBM group, showed a decrease in consumption of <15% over 41 days. The untrained GBM group exhibited a 16% increase in water and food intake.

### 3.3. Behavioral Parameters

**Figure 2:**
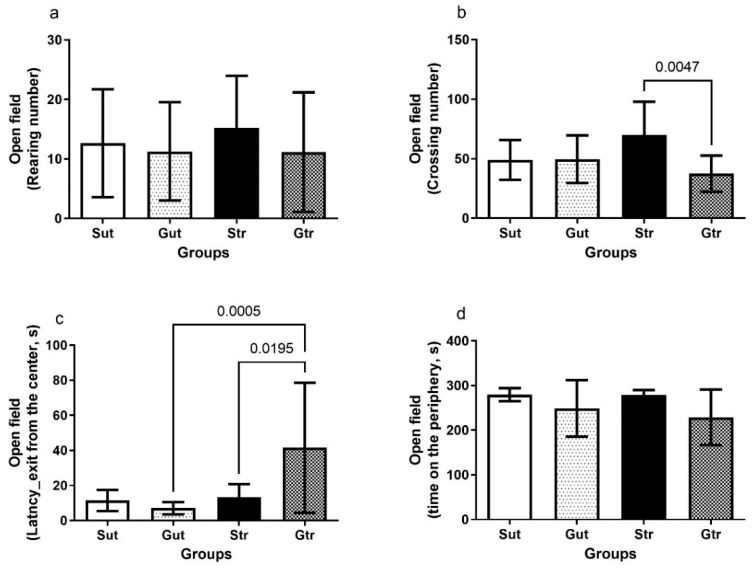
Open-field test of animals exposed to an experimental model of glioblastoma. Number of crossings (**a**), number of elevations (**b**), latency for exit from the center of the quadrant (**c**), length of stay in the periphery (**d**). Gut, untrained glioblastoma (*n* = 15); Gtr, trained glioblastoma (*n* = 12); Sut, untrained sham (*n* = 10); Str, trained sham (*n* = 10).

The results depicted in Figure 2 revealed the effects of physical training on the behavior of the experimental groups in the open-field test. The trained GBM group showed a reduced number of crosses compared to the STR group (*p* = 0.0047, 95% CI = 7.615 to 57.30; SE = 8.982) and higher latency to leave the center of the quadrant compared to the Gut (*p* = 0.0005; CI95% = −56.48 to −12.52; SE = 7.927) and Str groups (*p* = 0.0195; 95% CI = −53.31 to −3.194; SE = 9.038), indicating a difference in their exploratory behavior compared with the trained sham group. 

**Figure 3:**
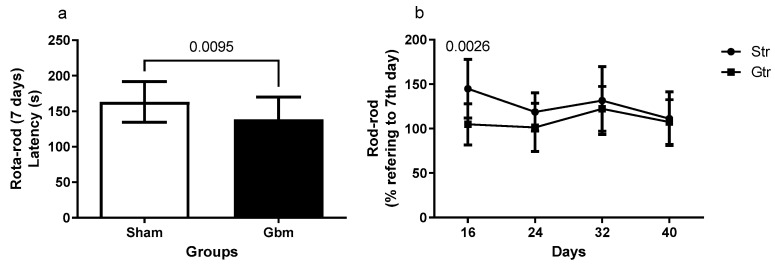
Rotarod of animals exposed to an experimental model of glioblastoma (GBM). Comparison of the rotarod between the sham and experimental groups 7 days after GBM induction (**a**), percentage of changes of the experimental groups in relation to the data of 7 days (**b**). Gut, untrained glioblastoma (*n* = 15); Gtr, trained glioblastoma (*n* = 12); Sut, untrained sham (*n* = 10); Str, trained sham (*n* = 10).

The data depicted in Figure 3 illustrate the results of the stretch bar test. The GBM group had a lower latency to fall 7 days after the initiation of surgical intervention compared with the sham group (*p* = 0.0095, 95% CI = −42.87 to −6.327) (Figure 3a). Upon comparison of the percentage changes in the latency of the trained groups, a statistically significant difference was detected only between the sham and GBM groups in the first week following GBM induction (*p* = 0.0026, 95% CI = 8.796 to 53.23; SE = 8.317), with no significant differences observed in the later stages (Figure 3b).

### 3.4. Production of Reactive Oxygen Species

**Figure 4:**
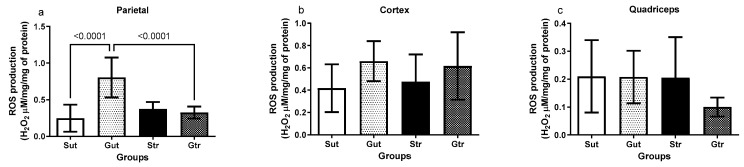
Analysis of reactive oxygen species (ROS) production of animals exposed to an experimental model of glioblastoma. Levels of ROS in the right parietal (**a**), cortex (**b**), and quadriceps (**c**). Gut, untrained glioblastoma (*n* = 15); Gtr, trained Glioblastoma (*n* = 12); Sut, untrained sham (*n* = 10); Str, trained sham (*n* = 10).

The data depicted in Figure 4 reveal an alteration in ROS production among the experimental groups. The animals with experimental GBM exhibited elevated levels of ROS in the parietal region compared with the sham group (Gut vs. Sut, *p* < 0.0001, 95% CI = −80.63 to −30.60; SE = 0.08812; Gtr vs. Gut, *p* < 0.0001; 95% CI = 0.2282 to 0.7286; SE = 0.8812), whereas both groups undergoing physical training showed significant reductions in ROS levels (Figure 4a). However, no changes were observed in the ROS production in the cortex (Figure 4b) and quadriceps (Figure 4c).

### 3.5. Oxidative Damage

**Figure 5:**
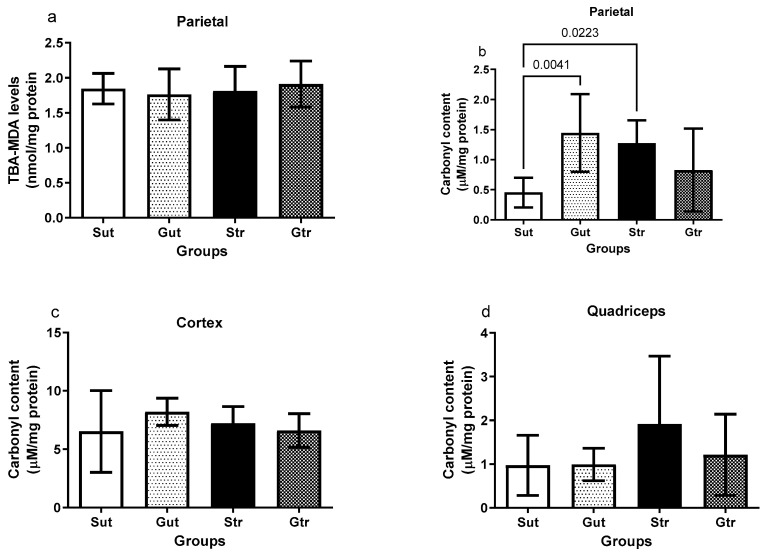
Oxidative damage of animals exposed to an experimental model of glioblastoma. Levels of lipoperoxidation in the right parietal (**a**) and carbonylation of proteins in the right parietal (**b**), cortex (**c**), and quadriceps (**d**). Gut, untrained glioblastoma (*n* = 15); Gtr, trained glioblastoma (*n* = 12); Sut, untrained sham (*n* = 10); Str, trained sham (*n* = 10).

The data presented in Figure 5 highlight the effects of experimental GBM and physical training on oxidative damage in the studied tissues. Although no significant changes in the MDA levels were observed in the right parietal region (Figure 5a), an increase in protein carbonylation was detected in the Gut and Str groups compared to the Sut group (Gut vs. Sut, *p*= 0.0041; 95% CI = −1.728 to −2.555; SE = 0.2586; Str vs. Sut, *p*= 0.0223; 95% CI = −1.557 to −0.0850; SE = 0.2586) The GBM-plus-exercise group remained similar to the sham group andshowed no significant difference in relation to the GBM group. No alterations in the carbonylation levels were observed in the cortex (Figure 5c) and quadriceps (Figure 5d).

### 3.6. DNA Damage

**Figure 6:**
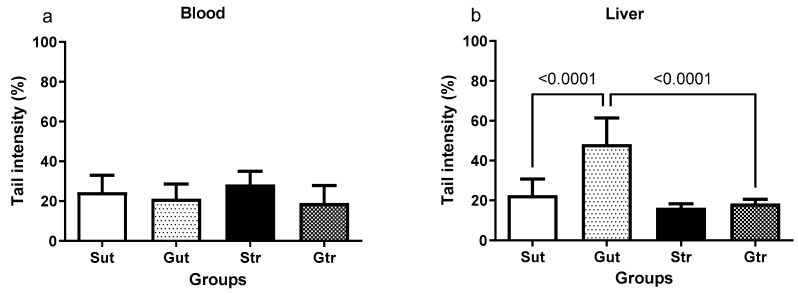
DNA damage parameters in the blood (**a**) and liver (**b**) of animals exposed to an experimental model of glioblastoma. Sut, untrained sham (*n* = 10); Gut, untrained glioblastoma (*n* = 15); Str, trained sham (*n* = 10); Gtr, trained glioblastoma (*n* = 12).

As shown in Figure 6, the tail intensity of the comet in the blood assay did not show significant differences among the experimental groups. However, the GBM group had significantly higher tail intensity in the liver (Figure 6b) compared with the sham group (*p* < 0.0001; 95% CI = −36.27 to −15.09; SE = 3.899), which was significantly reduced by physical training (*p* < 0.0001; 95% CI = 18.89 to 40.75; SE = 4.021).

### 3.7. Micronucleus

The results of the bone marrow micronucleus test in mice, as shown in Table 2, indicate that the GBM group had a significantly higher number of micronucleated polychromatic erythrocytes compared with the other groups (*p* < 0.05). However, no significant differences were observed in the proportion of EPCs/ENCs among the groups.

## 4. Discussion

GBM is an extremely aggressive brain tumor, the origin of which is the subject of intense debate, focusing on the theory of malignant transformation of neural or glial progenitor cells [7]. Current treatments, such as surgical resection followed by radiotherapy and chemotherapy, are necessary and only minimize disease progression but prolong life by a few months with the downside of reduced quality of life [19,20]. Therefore, it is important to understand the mechanisms of disease genesis and progression, modulated by nonpharmacological interventions that have the potential to be adjuvant to existing treatments, contributing to reducing morbidity and costs and, most importantly, improving patients’ quality of life.

Depending on the size and location of the tumor, cognitive and functional deficits are common in patients with GBM. Therefore, monitoring behavioral changes is of clinical significance in controlling and monitoring the disease. In the present study, the open-field and rotarod tests were used to evaluate anxiety, exploratory capacity, and locomotor activity [21]. The open-field test is relevant in studies that evaluate behavioral changes due to nervous system diseases, as it allows for the analysis of emotional and locomotor patterns in animals [22]. The results showed that the trained GBM group had lower exploratory capacity than the trained sham group, as revealed by the higher number of crossings. These results may be related to the natural tendency of non-diseased animals to explore new environments [23]. Moreover, the latency or exhaustion time obtained on the rotarod test was lower in the GBM group, and the trained sham and GBM animals showed a longer latency time compared with their respective controls at the end of the experimental period. These results demonstrate the important role of physical training in the regulation of locomotor parameters. Similar results using different experimental designs have been reported in previous studies [8,24]. Physical training promotes the release of neurogenic factors, such as brain-derived neurotrophic factor, in addition to regulating intracellular signaling pathways that inhibit neuronal dysfunction and promote neurodegenerative action. These neural processes contribute to the maintenance of locomotor and exploratory behaviors and to transient improvements in motor capacities [5,25,26].

Reactive oxygen species (ROS), including hydrogen peroxide (H_2_O_2_), can have varying effects dependent on the basal metabolic rate of cancerous cells, including those of Glioblastoma Multiforme (GBM) [27]. The Central Nervous System (CNS), given its high metabolic activity and rich concentrations of fatty acids and transition metals, is a significant facilitator of oxidant production [28,29,30]. Under normal circumstances, astrocytes and neurons are protected from oxidation by inherent antioxidant systems. However, under abnormal or stressed conditions, these cells can become susceptible to ROS-induced damage, leading to genetic instability and altered redox homeostasis [31]. In this context, it is important to consider the role of ferroptosis, a form of regulated cell death driven by iron-dependent lipid peroxidation. This process is intrinsically linked with oxidative stress and ROS. During ferroptosis, iron accumulation increases ROS production, especially lipid ROS, leading to lipid peroxidation and cell death [32]. This concept is particularly relevant when analyzing data related to ROS production and oxidative damage, given the intricate relationship between these factors and ferroptosis. Nonpharmacological therapies that aim to regulate the redox balance should work to alleviate the nonspecific signaling of ROS, while also promoting the reduction of oxidative processes, such as lipoperoxidation—a process crucially linked with ferroptosis [33]. The sensitivity of the brain to oxidative stress negatively impacts the levels of proteins involved in metabolism and cerebral neuroprotection [26,34]. H_2_O_2_ is observed in cells during cellular metabolism. It behaves similarly to the signals generated by Ca^2+^ amplified by kinase cascade activation or can be transmitted over long distances by conversion to more stable species, such as lipid peroxides, and has high concentrations in the extracellular environment. These signaling events are coupled with metabolism, phosphorylation cascades, transcriptional regulation, cytoskeletal rearrangements, cell replication, and other critical cellular functions [35,36,37]. Owing to these factors, evaluating the capacity for oxidant production, such as H_2_O_2_, has been an important strategy to understand the role of ROS on brain cancer metabolism [38,39]. The results of this study showed an increase in event-related oscillation levels in the parietal region of animals with GBM, with a significant reduction after the physical training program. These differences were not observed in other brain and peripheral tissues, such as the cortex and muscle. The increase in oxidant levels in the parietal region has a possible relationship with the location where the tumor develops, which is directly related to carcinogenic factors associated with mitochondrial redox alterations, increased nicotinamide adenine dinucleotide phosphate oxidase activity, and angiogenic phases, in addition to low levels or selective inactivation of the antioxidant mechanism present in carcinogenic tissues [40]. In experimental models of GBM, increased H_2_O_2_ levels play an important role in the formation of chronic brain tissue inflammation [41]. These effects on ROS production involve different signaling cascades that induce mutations and tumor progression. The effects of physical training on oxidant levels may be localized, because different regions of the brain have different antioxidant capacities and oxidative statuses [26]. These findings highlight the need for further studies to understand the role of physical activity in the redox status of brain cancer and its potential implications for new therapeutic approaches.

According to Pinho et al. [42], regular exercise can promote a synergistic effect between the muscles and brain, regulate gene expression, and induce molecular changes that lead to a more balanced redox environment, reducing the risk of neurodegenerative diseases. An increase in cellular oxidant levels without an antioxidant response, which establishes a redox balance, leads to oxidative damage that plays a cytotoxic role and promotes cell death [38]. The oxidative damage studied in this study was the carbonylation of amino acid residues, which is defined as an irreversible posttranslational modification that produces a carbonyl group on a protein. “Carbonyl stress” is characterized by the accumulation of reactive carbonylated molecules that confer increased reactivity to nucleophilic substrates, triggering biomolecular malfunction, increased toxicity, and cell death [43,44,45]. Carbonylated proteins are involved in various deleterious processes, such as inflammation and deficient immune responses. Hardiany et al. [46] have suggested an association between carbonylation levels and brain tumorigenesis in humans. Our results showed that animals with GBM had higher carbonylated protein content, and these values were significantly reduced after the physical training program. Notably, submaximal aerobic exercise results in a significant increase in protein carbonylation, with the effect of maintaining this increase for a few days, which can be attributed to the increase observed in the trained sham animals. However, when cells and tissues are under constant adverse stimuli, the long-term effects of physical exercise begin to modulate carbonylation levels by stimulating a reduction in the production of oxidants or increasing the tissue’s antioxidant protection [47]. Recent studies have shown similar effects of physical training on animal protein carbonylation in tumors. For example, Assi et al. [25] demonstrated that forced treadmill exercise reduced prostate cancer growth in rats by reducing oxidative damage. No significant changes were observed in the carbonylation levels in the cortex and quadriceps, suggesting that carbonylation may be tissue-dependent on injury, as tumor presence occurs in specific brain areas.

Cancer is characterized by genomic instability with alterations, such as copy number changes, sequence alterations, DNA methylation, and chromosomal rearrangements, which are hallmark features of cancer cells that promote tumor growth and progression [48]. The results of this study suggested that the GBM group had higher tail intensity in the liver in the comet assay compared with the trained groups, and these results were reversed with physical training. Observations made using the comet test suggest that genomic injuries can lead to mutations [49,50,51]. Our results showed that the group that received GL261 cells and did not exercise had higher tail intensity in hepatocytes compared with the trained sham and GBM groups, indicating greater DNA damage, which has been previously observed in studies that associate comet assay and physical exercise. Groups that exercise have lower DNA damage compared with groups that do not exercise [52,53].

MN are expressed in dividing cells that contain chromosomal breaks without centromeres (acentric fragments) and/or in entire chromosomes that are unable to migrate to the poles during mitosis. Malfunction of centromeres or kinetochores of chromosomes, or mitotic damage, can lead to the loss of entire chromosomes (aneuploidy) [54,55,56]. We evaluated the presence of MNs in the bone marrow of mice and observed that the animals with GBM had a significantly higher number of polychromatic erythrocytes with MNs compared with the sham group, and this increase was even higher in the GBM-trained animals. In previous studies [57], therapeutic intervention is effective in reducing DNA damage, resulting in a lower number of polychromatic erythrocytes with micronuclei (EPCMn). In our study, GBM increased the EPCMn content, but therapeutic intervention reduced the amount of EPCMn in the trained GBM group, indicating that physical training potentially mitigates the adverse effects of cancer. Future studies should focus on pinpointing the most effective exercise protocols for Glioblastoma Multiforme (GBM) patients and assess the clinical impact of physical activity on improving a variety of outcomes. In keeping with this, a few ongoing clinical trials are currently investigating the influence of exercise. Specifically, one trial (NCT03390569) is delving into the potential of exercise routines to enhance progression-free survival, overall survival, and quality of life for glioblastoma patients. Simultaneously, another study (NCT03775369) is focused on the effect of physical activity on the quality of life and overall well-being of individuals diagnosed with high-grade gliomas.

## 5. Conclusions

Overall, our results demonstrate that physical exercise modulates behavioral, redox, and DNA damage in animals with experimental GBM. These findings indicate that physical exercise potentially enhances brain function and reduces oxidative stress and genomic instability, which are associated with the development and progression of GBM. This, in turn, may lead to improvements in the cognitive function and quality of life of patients with GBM. However, it is important to highlight that the efficacy of the physical exercise observed in this animal experiment should be validated in future human studies. The type, intensity, and duration of physical exercise for patients with GBM should be further investigated. Taken together, physical exercise shows promise as an adjunctive therapy for the management of GBM by modulating redox parameters in the parietal tissue and reduces the genomic instability in liver and blood. 

## Figures and Tables

**Table 1:** Data on water consumption and food intake of the animals during the experimental period.

Groups	Average Water Consumption (Mean ± Standard Deviation)			% Change *	Average Food Intake (Mean ± Standard Deviation)			% Change *
	Post-Surgery	Day 7	Day 48		Post-Surgery	Day 7	Day 48	
Sut	2.8 ± 0.9	4.8 ± 0.9	4.7 ± 1.1	−1.3	2.7 ± 0.2	4.8 ± 0.8	4.7 ± 1.1	−1.3
Gut	1.1 ± 0.3	3.1 ± 1.1	3.5 ± 0.1	16.5	1.1 ± 0.3	3.0 ± 0.9	3.5 ± 0.2	16.6
Str	1.6 ± 0.9	5.1 ± 0.5	4.3 ± 1.3	−14.7	1.6 ± 0.9	5.0 ± 0.5	4.3 ± 1.1	−14.3
Gtr	1.0 ± 0.4	3.9 ± 0.1	3.3 ± 0.0	−13.7	0.9 ± 0.1	3.7 ± 0.1	3.4 ± 0.1	−8.1

Data are expressed as average consumption per box ± standard deviation; Gut, untrained glioblastoma (*n* = 15); Gtr, trained glioblastoma (*n* = 12); Sut, untrained sham (*n* = 10); Str, trained sham (*n* = 10). * Between aged 7 and 48 days.

**Table 2:** Analysis of micronuclei in the medulla of animals exposed to an experimental model of glioblastoma. A total of 2000 cells per sample were analyzed. Two-way analysis of variance followed by the Bonferroni test was performed considering a *p* value < 0.05. The data are expressed as mean ± standard deviation.

Groups	MN-PCE	MN-MCE	PCE/NCE
Sut	2.71 ± 1.11	0.71 ± 1.11	0.56 ± 0.05
Str	3.25 ± 0.95	1.57 ± 1.81	0.50 ± 0.05
Gut	4.33 ± 1.03 *	1.10 ± 1.37	0.52 ± 0.05
Gtr	6.57 ± 1.90 ^#&^	1.90 ± 1.85	0.56 ± 0.05

* Significant difference compared with the Sut; # Significant difference compared with the Str; & Significant difference compared with the Gut. MN-PCE, micronucleus polychromatic erythrocytes; MN-MCE, micronucleus normochromatic erythrocytes; Sut, untrained sham (*n* = 8); Gut, untrained glioblastoma (*n* = 8); Str, trained sham (*n* = 8); Gtr, trained glioblastoma (*n* = 8).

## Data Availability

All datasets generated and analyzed in the current study are available from the corresponding author upon reasonable request.
